# Getting the Lead Out of Electronics

**DOI:** 10.1289/ehp.113-a682

**Published:** 2005-10

**Authors:** Harvey Black

The electronics industry is learning to do without: it is having to abandon one of its long-time staples, lead–tin solder. For decades lead–tin solder has been used to attach electronic components to printed wiring boards. However, with the body of evidence pointing to serious adverse health effects of lead, the search for a replacement has spawned intense effort in the electronics industry and in universities. Now scientists think they may have found some promising leads: solders made of alternative alloys and polymer formulations known as electrically conductive adhesives (ECAs).

## The Linchpin of Electronics

Solder is the “linchpin of electronics manufacturing,” says Jack Geibig, acting director of the Center for Clean Products and Clean Technologies at the University of Tennessee. “Without it, it’s difficult to achieve a proper electronic connection that is durable and reliable.”

Lead has been ideal for solder. In fact, says Carol Handwerker, chief of the metallurgy division at the National Institute of Standards and Technology, “The whole electronics infrastructure was designed around the melting point and physical properties of [lead].” Lead is malleable and thus easy to work with, and it doesn’t fracture, she says. When lead is combined with tin in the correct proportion (63% tin to 37% lead), the resulting alloy has a low melting point of 183°C, which is another advantage, Geibig says: “If you’re not operating at really high temperatures, you have more control over processes, so that the processes aren’t sensitive to slight temperature variations, which are costly to control.” Low temperatures also mean less strain on the equipment and materials (such as printed circuit board and components) that must be heated as part of the assembly process.

The main impetus for the industry to leave lead behind is a ban on lead in electronics imposed by the European Union. Under the Restriction of Hazardous Substances directive, as of 1 July 2006 lead must be replaced by other substances in electronic equipment. (The directive also bans mercury, cadmium, and hexavalent chromium.) Any electronic components bound for Europe are subject to the ban.

Lead is not a problem when contained in electronic equipment, says Robert Donkers, an environmental counselor for the European Commission who is based in Washington, DC. However, when electronic components are deposited in landfills, he says, people may scavenge for equipment and break it open, or the lead may leach out of landfills and into drinking water. The risk is compounded in countries that receive massive imports of electronic waste. In China, for example, unprotected workers, including many children, strip recyclables out of electronic components in a cottage industry of sorts [see “e-Junk Explosion” in the April 2002 issue of *EHP*].

Lead exposure, even at low levels, is well known for its harmful effects on children, resulting in lowered IQ. Lead also affects the ability to pay attention. Children exposed to low levels may appear hyperactive and irritable, according to the American Academy of Child and Adolescent Psychiatry. The current maximum allowable level for blood lead in the United States is 10 micrograms per deciliter (μg/dL).

## Alternative Alloys

The main approach to replacing lead in solder has been to look for other metals as substitutes. Electronics manufacturers began to look for alternative metals in the 1990s, notes Handwerker, when now-abandoned proposals were being discussed in the United States to ban lead in electronics.

Ronald Gedney, a consultant for the International Electronics Manufacturing Initiative (iNEMI), a technology consortium, has been intimately involved in the search for alternatives. He says that a search by industry experts for possible replacements for lead–tin solder winnowed down 75 metal alloy alternatives to about half a dozen. “We decided the biggest benefit for the industry would be to pick one solder, concentrating our development and research efforts on one alloy and making it work,” he says.

The industry eventually selected a tin–silver–copper combination as offering the most reliability and ease to work with as a replacement. The formulation—95.5% tin, 3.9% silver, 0.6% copper—is also known as SAC solder, for the first letters of the chemical symbols of each of the elements (Sn, Ag, Cu). “Tin–silver–copper appears to have at least as good reliability if not higher reliability than tin–lead,” says Handwerker.

Furthermore, according to a 2005 draft report issued by the U.S. Environmental Protection Agency titled *Solders in Electronics: A Life-Cycle Assessment*, silver was “rarely encountered above the detection limit” in synthetic landfill leachate created to test the stability of electronics components. Silver—which is regulated as a hazardous material—is toxic to aquatic life.

With a melting point of 217°C, SAC solder also is closest in melting point to the conventional lead–tin solder. This does mean, however, a yet-unquantified increase in energy use. Furthermore, the higher temperature may pose problems for the electronics industry. Higher temperatures mean more stress on components and the entire manufacturing process, notes Geibig. Higher temperatures also mean increases in the time it takes to make products, because more time is required to heat and cool the products during the course of their manufacture.

SAC solder is used widely in the industry today. However, many of the components being made could not withstand the higher temperatures, says C. Michael Garner, director of materials technology operations at Intel: “That required re-engineering and getting new materials, not only for newer products but for older products. All the older products that had been in production for ten or fifteen years had to be converted over to high temperatures.” He says it has taken a massive effort to integrate the new solder into production processes.

There are also short-term consequences of using the new solder. Anytime there is a change in materials, there is a learning curve in using the new materials, says Karl J. Puttlitz, who managed IBM’s efforts to reduce lead in its products before he retired last year. He anticipates the occurrence of more manufacturing defects as a result of the changeover. “We can expect that at least initially the failure rates [of products] will increase,” he says. In fact, he notes the industry has asked for exemptions to the EU lead ban in certain critical electronic components where lives and security might be involved, such as equipment used in hospitals, until a track record is established with consumer goods such as cell phones and digital cameras. (The EU directive does permit exemptions to the lead ban if replacing lead is technically or scientifically impractical or if negative health, environmental, or safety consequences of replacing lead outweigh the benefits of the ban.)

## A Stickier Approach

A more experimental alternative to lead–tin solder is the use of ECAs. These are polymers, such as silicone or polyamide, containing tiny flakes of metals such as silver. The polymers adhere to the printed circuit boards, and the metal flakes conduct electricity.

ECAs offer a range of advantages, notes C.P. Wong, a professor in the School of Materials Science and Engineering at the Georgia Institute of Technology who is regarded by many in the field as the leading researcher in this new technology. Silver’s electrical conductivity is very high, and its electrical resistance is very low, he points out. “If the current-carrying capability [can be boosted], ECAs can replace solder,” he says.

And there is another benefit. The temperature required to apply ECAs to circuit boards is far lower than that required for lead-based solder—150°C compared to 183°C. “You save energy, number one,” says Wong. “Number two, you subject all the components to lower temperatures and thus less thermomechanical stress. That enhances their reliability.”

Preliminary studies comparing parts using ECAs instead of solder, such as a Finnish study presented in 2000 at the 4th International Conference on Adhesive Joining and Coating Technology in Electronics Manufacturing, suggest that ECAs boast a much tighter bond than solders—perhaps an order of magnitude better, says James Morris, a professor of electrical and computer engineering at Portland State University. But he adds this research has to be replicated before it is regarded as valid.

ECAs are available for a small number of applications requiring low power—for instance, liquid crystal displays—though they are not ready for the marketplace in general, where greater amounts of current are needed. Wong is working to enhance their ability to carry current. He is adding molecules of dicarboxylic acid to the silver flakes, which provides a link between the flakes, allowing for efficient and quick conduction of electric current. “It looks like it can be as good as or even better than lead–tin solder. We demonstrated that it works [in a presentation at the March 2005 national meeting of the American Chemical Society], but we still need further research and development,” says Wong.

Wong and his collaborators are also using another means to boost the capacity to carry current—self-assembled monolayers. These are single layers of sulfur-containing molecules known as thiols that are attached to gold pads in the electronic device. At less than 10 angstroms (10 ten-billionths of a meter) in length, the molecules chemically bind to the gold pads in the device and the board, providing a direct electrical connection.

Still more work is needed on these structures, however, because they begin to fail structurally if the component heats up above 150°C. And there are other concerns about ECAs. With time, notes Wong, the ability of ECAs to conduct electricity drops, and resistance to electricity increases. Another concern is moisture. “Water is absorbed by polymers, in general,” says Morris. That can encourage corrosion, he says, and may cause other as yet unknown problems, he says.

Wong also points to the need for ECAs to become tougher so they can withstand the force of being dropped. One way to do this, says Wong, is to develop polymers that are rubberized and made more elastic, so they won’t break. Finally, Garner reiterates that these materials have not been reliable for carrying moderate to high amounts of current under normal operating conditions.

Wong and Morris are optimistic that with more research and development, ECAs can be successful alternatives to lead–tin solder. And Puttlitz does see a place for them in consumer electronics such as cell phones and digital cameras, which are not “mission critical” applications where reliability is a matter of life and death as in medical monitoring equipment or aircraft electronics.

## Solder Replacement Soldiers On

Even as efforts to replace lead in solder move ahead, there still appear to be concerns about the impact that newly implemented metals will have on human and environmental health. “The alternatives to lead have not been researched as well as lead in terms of potential health and environmental impacts,” says Oladele A. Ogunseitan, a professor of environmental health, science, and policy at the University of California, Irvine. “When the Europeans said industry must get rid of lead, they didn’t say you must replace lead with something that is obviously safer,” he notes wryly. It is important, he adds, to keep looking for lead alternatives that are environmentally benign.

Indeed, the draft *Solders in Electronics* report indicates that no metallic alternative to lead is free from environmental concerns. For instance, whereas lead may pose a greater public health problem than SAC solder, the latter uses noticeably more energy than lead–tin solder.

But the presence of today’s substitutes is good enough for Donkers. “Since there are alternatives, we have chosen not to have lead in the products anymore,” he says. And while he does acknowledge that there are relatively few data on the impact of the current lead solder alternatives, he asserts that “in terms of active policy, you cannot always wait till you have complete certainty, because in the meantime a lot of people get exposed [to lead].”

## Figures and Tables

**Figure:**
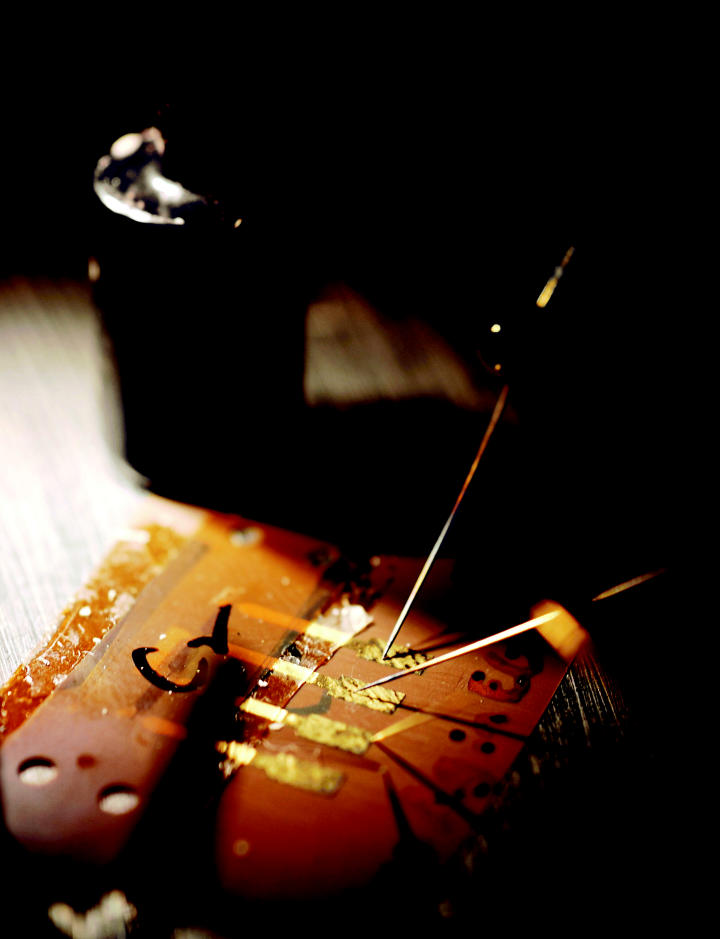
Sticking with the problem. Electrically conductive adhesives are one alternative to lead–tin solder being tested in the search for healthier electronics.

**Figure:**
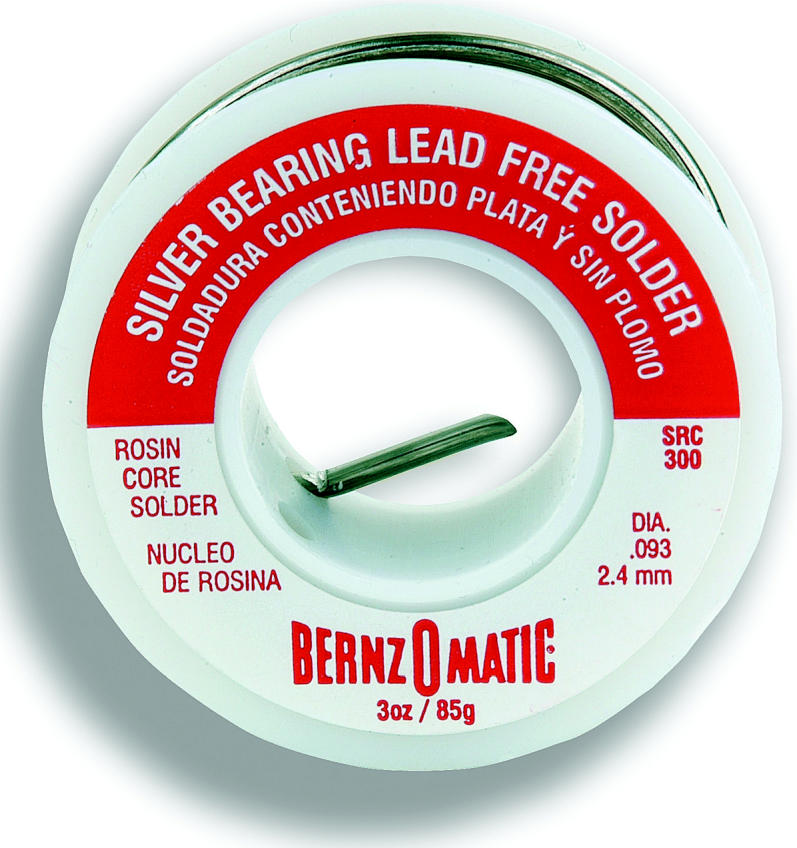
The alloy alternative. Tin–silver–copper solder offers a safer solder than the lead–tin alloy, and research is continuing to address limitations on its use.

**Figure:**
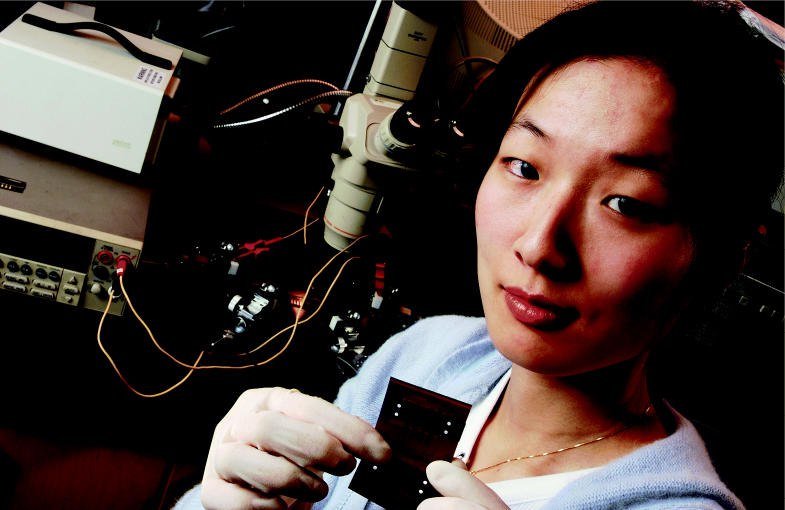
Current advances. Researcher Grace Yi Li holds samples of electrically conductive adhesives being studied at Georgia Tech’s School of Materials Science and Engineering. Such adhesives may one day replace lead-based solders.

## References

[b1-ehp0113-a00682] Geibig JR Socolof ML 2005. Solders in Electronics: A Life-Cycle Assessment (Draft). Environmental Protection Agency, Office of Pollution Prevention and Toxics. Available: http://www.epa.gov/dfe/pubs/solder/lca/index.htm

[b2-ehp0113-a00682] Li Y, Moon K, Wong CP (2005). Electronics without lead. Science.

[b3-ehp0113-a00682] Puttlitz KJ Stalter KA eds. 2004. Handbook of Lead-Free Solder Technology for Microelectronic Assemblies. New York, NY: Marcel Dekker.

[b4-ehp0113-a00682] Schoenung JM, Ogunseitan OA, Saphores J-DM, Shapiro AA (2004). Adopting lead-free electronics: policy differences and knowledge gaps. J Ind Ecol.

